# SYNCAS‐mediated CRISPR‐Cas9 genome editing in the Jewel wasp, *Nasonia vitripennis*

**DOI:** 10.1111/imb.70002

**Published:** 2025-07-17

**Authors:** Filippo Guerra, Sander De Rouck, Eveline C. Verhulst

**Affiliations:** ^1^ Laboratory of Entomology, Plant Sciences Group Wageningen University Wageningen The Netherlands; ^2^ Laboratory of Agrozoology, Department of Plants and Crops, Faculty of Bioscience Engineering Ghent University Ghent Belgium

**Keywords:** BAPC, gene editing, *Nasonia vitripennis*, saponin, SYNCAS

## Abstract

Genetic engineering is a formidable approach to studying biology. The development of CRISPR‐Cas9 has allowed the genetic engineering of insect species from several orders, and in some species, this tool is used routinely for genetic research. However, insect gene editing often relies on the delivery of CRISPR‐Cas9 components via embryo injection. This technique has a limitation: some species lay their eggs inside hard substrates or living hosts, making embryo collection impossible or labour intensive. Recently, a variety of techniques that exploit maternal injection of nucleases have been developed to circumvent embryo injection. Yet, despite this variety of maternal delivery techniques, some insects remain refractory to gene editing. One of these is the parasitoid wasp, *Nasonia vitripennis*, an important hymenopteran model species. In this study, a recently developed method termed SYNCAS was used to perform knock‐out (KO) of the *cinnabar* gene in this wasp, obtaining KO efficiencies up to 10 times higher than reported for other maternal injection approaches. We found up to 2.73% of all offspring to display a KO phenotype, and we obtained up to 68 KO offspring per 100 injected mothers. The optimal timing of injection and provision of hosts for egg laying was determined. With this protocol, routine applications of CRISPR‐Cas9 become feasible in this species, allowing reverse genetics studies of genes with unknown associated phenotypes and paving the way for more advanced editing techniques.

## Graphical abstract

- Maternal injection of Cas9 RNP together with BAPC and saponins (SYNCAS) results in efficient gene editing of *Nasonia vitripennis* offspring.
- The timing of injection and egg collection is important for maximising efficiency and reducing screening effort.
- The efficient genetic engineering of *Nasonia vitripennis* suggests the applicability of SYNCAS to other parasitoid wasps, a category of insects particularly difficult to genetically engineer with embryonic Cas9 injections.

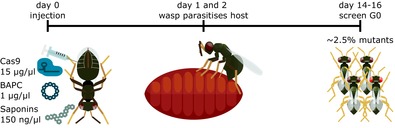

## INTRODUCTION

The remarkable diversity of insects presents both an intriguing subject of study and a significant challenge for the development of standardised research methodologies. In particular, the difficulty in obtaining gene‐edited insects frustrates entomologists just as much as it surprises drosophilists. Indeed, thousands of *Drosophila* lines are available, yet many species of insects remain refractory to being genetically engineered. Recent advances, including the implementation of CRISPR‐Cas9‐based approaches, have partially addressed these challenges. CRISPR‐Cas9 relies on a Cas9 nuclease directed by a guide RNA to induce targeted DNA modifications, offering a powerful tool for genome editing. The Cas9 ribonucleoprotein (RNP) complex is usually injected in early embryonic stages, before the onset of nuclear division (Burger et al., 2016). However, embryos are not always readily accessible, and microinjections can cause high lethality. This is true for parasitoid species that lay their eggs inside living hosts, species that lay eggs into hard substrates, or species that lay minuscule eggs.

To circumvent these limitations, researchers developed alternative approaches that bypass direct embryonic manipulation and rely on maternal injection of CRISPR‐Cas9 components to target the offspring. Some species are efficiently modified upon maternal injections of Cas9 RNP alone, a technique termed direct parental (DIPA) CRISPR. Insects as diverse as *Blattella germanica*, *Tribolium castaneum* (Shirai et al., 2022), *Aedes aegypti* (Shirai et al., 2023), *Sogatella furcifera* (Zhang et al., 2024a) and *Orius strigicollis* (Matsuda et al., 2024) are efficiently modified using DIPA CRISPR. For other species, the use of Cas9 fused with a yolk‐protein‐derived peptide that triggers receptor‐mediated uptake by the ovaries works best. This technique, termed Receptor‐Mediated Ovary Transduction of Cargo (ReMOT Control), was used for the modification of mosquitoes (Chaverra‐Rodriguez et al., 2018; Macias et al., 2020), *Bombyx mori* (Yu et al., 2023), *Diaphorina citri* (Chaverra‐Rodriguez et al., 2023), and *Rhodnius prolixus* (Lima et al., 2024). A drawback of this method is that the yolk‐peptide sequence is, to some extent, species‐specific, and may necessitate the production of tailor‐made Cas9 depending on the target organism (Chaverra‐Rodriguez et al., 2018; Heu et al., 2020). Another variation on DIPA uses chemicals to enhance Cas9 uptake by the oocytes; branched amphipathic peptide capsules (BAPC) have been used in the psyllid *D. citri* to reduce the surface charge of the RNP complex and promote its ovarian uptake (Hunter et al., 2018).

Despite the versatility and ease of use of the techniques based on maternal injection, they are no silver bullet: in the hymenopteran model *Nasonia vitripennis*, DIPA, REMOT and BAPC‐assisted Cas9 delivery were shown to work (Chaverra‐Rodriguez et al., 2020; Zhang et al., 2024b) but the obtained efficiencies are insufficient to be used in practice (Table 1).

**TABLE 1:** Efficiencies of previously published techniques for *Nasonia vitripennis* genome editing.

Technique	Injection	Number of injections	Wasps screened	GM wasps obtained	Reference
TALEN	Embryonic	Unknown	NA	Unknown	Lynch (2015)
CRISPR‐Cas9	Embryonic	100	20 (all those surviving)	12 (60%)	Li et al. (2017)
DIPA	Maternal	179	4970	4 (0.08%)	Zhang et al. (2024b)
REMOT	Maternal	121	1229	4 (0.3%)	Chaverra‐Rodriguez et al. (2020)
BAPC	Maternal	60	943	8 (0.8%)	Chaverra‐Rodriguez et al. (2020)

*Note*: Although a direct comparison between techniques is not possible, the values are indicative of the drawbacks of each technique: embryonic injections cause high mortality and are laborious to perform, maternal injections require the screening of thousands of offspring.

Several aspects of *Nasonia* biology make it a highly convenient laboratory study system: it parasitises blowfly pupae that are easily available as maggots for fishing, it has a short biological cycle (10 days at 28°C, 14 days at 25°C and 21 days at 20°C) (Werren & Loehlin, 2009), and diapause can be easily induced, creating “biological backups” (Saunders, 1966). Moreover, due to the haplodiploid nature of *Nasonia vitripennis* sex determination, virgin females lay only eggs which develop into haploid males. This eases the screening of mutants as KO events always produce an effect on the phenotype, even when the mutation is recessive, and simplifies the establishment of homozygous mutated lines (Lynch, 2015).

Unfortunately, genetically engineering *Nasonia* proved exceptionally difficult. First attempts relied on embryo microinjections which are laborious, require micromanipulators and result in massive embryo lethality (Li et al., 2017; personal observations). Moreover, the high efficiency obtained by Li et al. (2017) (Table 1) could not be replicated, as shared by many researchers at the last International *Nasonia* meeting (28 July 2023, Münster, DE). Subsequent efforts exploited maternal injections with REMOT technology and the use of BAPC as adjuvants (Chaverra‐Rodriguez et al., 2020), and finally, DIPA (Zhang et al., 2024b), but the low efficiency makes screening for mutant offspring laborious.

Recently, a technique named SYNCAS was developed to engineer arthropods with off‐the‐shelf nucleases and chemicals (De Rouck et al., 2024; Mocchetti et al., 2025). SYNCAS relies on, and is named after, the synergism between a surfactant (saponins) and nano‐capsules (BAPC) to aid protein uptake in a poorly understood, yet efficient manner. We applied the technique to KO *cinnabar*, a gene responsible for eye colour, in *N. vitripennis*. We optimised the timing of injection in the adult female, the timing of provision of fly pupae (hosts) for egg laying, and the concentration of saponins. With this optimised protocol, we reached an efficiency of 2.73% offspring with mutated phenotype and a maximum of 67.9 mutant wasps per 100 maternal injections, which is significantly higher than previously reported studies.

## RESULTS

### Saponin toxicity

**FIGURE 1:**
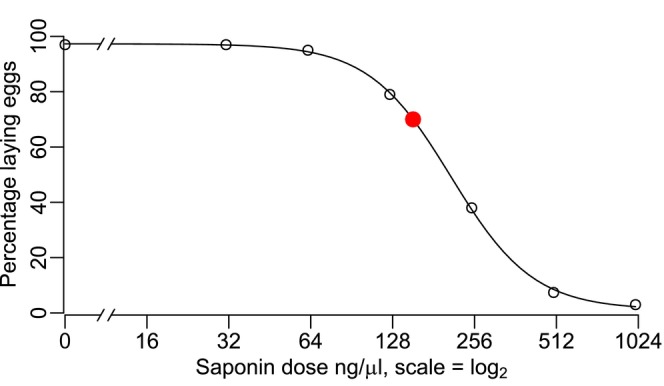
Dose–response curve for saponin effect on *Nasonia vitripennis* egg‐laying. The percentage of egg‐laying females falls steeply with concentrations of saponins between 120 and 350 ng/μL. The estimated dose that would allow for 70% egg‐laying ability is 150 ng/μL, and is reported as a red dot. White dots represent measured values, also reported in Table S1. Dose–response curve and estimated dose were calculated with the R package drc (Ritz et al., 2015).

Previous studies reported that endosomal escape is dependent on saponin concentration (Cao et al., 2020), suggesting that saponin concentration and maternally provided Cas9 efficiency could positively correlate (Macias et al., 2020). However, this chemical is toxic to *Nasonia* (Chaverra‐Rodriguez et al., 2020), and a trade‐off between mother survival and gene editing efficiency must be accounted for. We injected saponins in a range between 0 and 1 μg/μL to define the maximum amount of saponins that would allow 70% of the wasps to lay eggs. A dose–response curve was fitted to the data (*R* ^2^ = 0.99) (Figure 1; source data in Table S1). Based on these results, we estimated that injections with saponins at a concentration of 150 ng/μL would result in 70% of females laying eggs. Therefore, this concentration was used as the standard for the SYNCAS formulation in *N. vitripennis*.

### Saponins concentration affects SYNCAS efficiency

**FIGURE 2:**
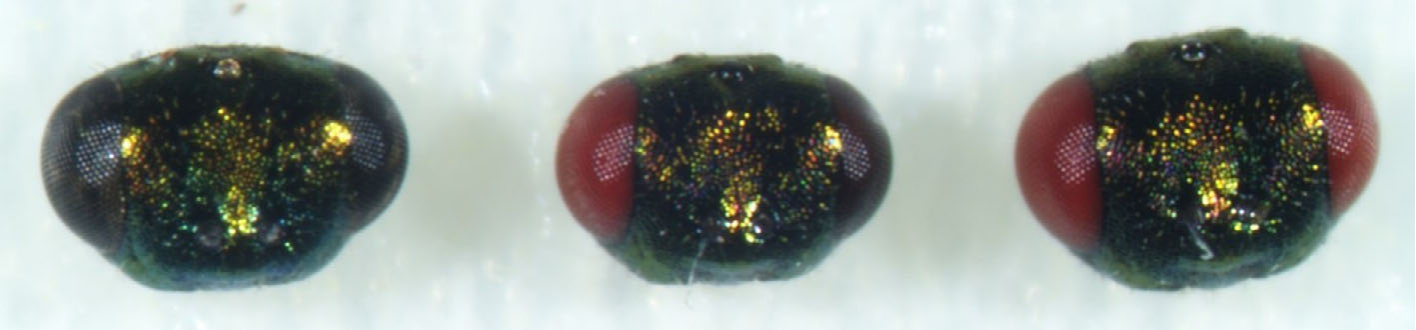
Phenotypes of G0 offspring after SYNCAS treatment. From left to right, frontal view of the head of a male with wildtype dark eyes, the head of a chimeric male having a wildtype and a red eye, and a male exhibiting the mutant phenotype, red eyes.

We tested three concentrations of saponins (0, 150, and 300 ng/μL) to evaluate their impact on gene‐editing efficiency, leaving the dose of Cas9, sgRNA and BAPC constant. These formulations were injected into 1‐day‐old virgin females. To identify the time window when most genetically engineered eggs are laid, we collected parasitised hosts three times, defining three time windows: between 0 and 24 h, between 24 and 48 h and over 48 h post‐injection. Offspring were screened for the red‐eye phenotype shown in Figure 2. Following our previous result, saponin dose impacted survival as well as egg‐laying capability and had a great impact on the production of genetically engineered offspring (Table 2).

**TABLE 2:** Effects of saponin concentration on *Nasonia vitripennis* history traits and SYNCAS efficiency.

Saponin concentration [ng/μL]	Timepoint [hours]	Surviving females	Females producing offspring	Females producing GM offspring	Total number of offspring	GM offspring (of which chimeras)	% GM
0	0–24	51/51	45/51	1	1428	1	0.07
0	24–48	50/51	50/51	1	2030	1 (1)	0.05
0	48‐	50/51	50/51	0	3110	0	0
150	0–24	53/56	32/56	5	403	11	2.73
150	24–48	53/56	51/56	11	1204	27	2.25
150	48‐	53/56	51/56	0	2764	0	0
300	0–24	19/45	12/45	0	119	0	0
300	24–48	15/45	11/45	4	195	5	2.56
300	48‐	15/45	13/45	1	371	1	0.27

*Note*: Saponins aid the genetic engineering of *N. vitripennis* embryos, yet have a detrimental effect on the survival of injected females. A concentration of 150 ng/μL resulted in more than 2% genetically modified (GM) offspring over a 48‐h‐long period. The total number of offspring for each treatment and time interval was estimated by multiplying the average number of wasps in 12 randomly selected tubes by the total number of females producing offspring.

We calculated efficiency with two parameters: the number of mutants obtained per mother, and the percentage of mutants of the total number of offspring. Injections without saponin in 51 wasps yielded two mutant males (0.04 mutants per wasp injected) of which one had a chimeric phenotype. Injections in 56 wasps with 150 ng/μL yielded 38 mutant males (0.68 mutants per wasp injected), and injections in 45 wasps with 300 ng/μL yielded six mutant males (0.13 mutants per wasp injected). When calculating efficiency in relation to the number of offspring, injections yielded a maximum of 0.07%, 2.73% and 2.56% GM offspring when injecting 0, 150 or 300 ng/μL saponins, respectively. Additionally, we identified the first 48 h post‐injection as the time window when most genetically engineered eggs are laid, highlighting the importance of timing in increasing the efficiency of the technique. This raised the question of whether injecting wasps at a different age or timing of host provision would affect efficiency.

### Injection and host provision timing both affect SYNCAS efficiency

We performed two additional injection rounds using the optimal saponins concentration of 150 ng/μL. One injection was performed in four‐day‐old adult females, a time when ovogenesis is in full swing (personal observations). The second was performed in one‐day‐old adult females (as in the previous round of injections), but with host provision being delayed by 24 h to allow for a longer activity of the injected components on the developing eggs. Genetically engineered offspring were produced in high numbers in all conditions, yet neither per‐offspring nor per‐mother injected efficiency was improved (Table 3). Indeed, injections in 90 4‐day‐old wasps yielded 52 mutant males (0.58 mutants per wasp injected), while injections in 55 1‐day‐old wasps and postponing host provision yielded 27 mutant males (0.49 mutants per wasp injected). In both conditions, the highest proportion of genetically engineered offspring was laid in the second host provided, which was offered between 24 and 48 h post‐injection to 4‐day‐old wasps, and between 48 and 72 h post‐injection to 1‐day‐old wasps.

**TABLE 3:** The effect of injection and host provision timing on SYNCAS efficiency.

Wasp age [days]	Timepoint [hours]	Surviving females	Females producing offspring	Females producing GM offspring	Total number of offspring	GM offspring (of which chimeras)	% GM
One	0–24	53/56	32/56	5	403	11	2.73
One	24–48	53/56	51/56	11	1204	27	2.25
One	48‐	53/56	51/56	0	2764	0	0
Four	0–24	72/90	77/90	2	2895	3	0.10
Four	24–48	72/90	72/90	7	1915	18 (1)	0.94
Four	48‐	72/90	72/90	11	3780	31	0.82
One, delayed rehosting	24–48	50/55	41/55	5	778	6	0.77
One, delayed rehosting	48–72	47/55	43/55	6	1123	16	1.42
One, delayed rehosting	72‐	46/55	44/55	1	2174	5	0.23

*Note*: Changing the age of the wasp at injection time and postponing host provision did not improve the efficiency compared with the first round of injections, reported here again for ease of comparison. The total number of offspring was estimated as in Table 2.

### Chimerism and mutation heritability

To define the percentage of mutated alleles in G0 red‐eye males, we amplified via PCR a fragment of *cinnabar* encompassing the Cas9 target site, and then we pooled the amplicons per treatment and mass‐sequenced them using Nanopore technology. The raw data obtained is accessible at https://doi.org/10.6084/m9.figshare.28677017, along with a document reporting the prediction of the most prevalent gene‐editing event sequences.

Injections with no saponins resulted in two chimeric offspring and 50% of all alleles being mutated. Sequencing offspring deriving from injections with saponins showed that 83%–95% of the amplicons obtained from red‐eyed males were mutated (Table 4), and had in‐dels between +10 and −11 bp in size (Figure S1). As expected, the transmission of the mutation positively correlates with the proportion of mutated alleles detected in the mass sequencing.

**TABLE 4:** Percentage of mutated alleles and G2 offspring in different SYNCAS treatments.

Treatment	Mutated alleles (%)	G2 with red eyes (%)
One day old, no saponins	50*	0/2 (0)
One day old, 150 ng/μL saponins	95	28/30 (93)
One day old, 300 ng/μL saponins	84	5/6 (83)
Four days old, 150 ng/μL saponins	93	40/45 (89)
One day old, 150 ng/μL saponins, delayed host provision	95	22/23 (96)

*Note*: The percentage of mutated alleles was inferred from Nanopore sequencing, except for the treatment “One day old, no saponins”, where amplicons were individually sequenced with Sanger technology (*), revealing the chimeric nature of both individuals. The mutation heritability was inferred by the presence of G2 offspring with red eyes.

Moreover, we defined the heritability of the mutation by mating the G0 red‐eye males with wildtype females and allowing the resulting G1 virgin females to lay male G2 offspring. The presence of red‐eye G2 would then confirm the transmission of the mutation. The results, reported in Table 4, suggest that genomic engineering resulting from the use of saponins has higher heritability compared with those obtained by injections of Cas9 and BAPC alone. However, the reduced sample size does not allow for a definitive conclusion. Indeed, mutant wasps obtained from injections without saponins sired no mutant offspring, whereas mutants obtained from SYNCAS injections transmitted the mutation at rates between 83% and 96%. Different SYNCAS treatments (i.e., injections with saponins) do not differ in mutation transmission rate [*X* ^2^ (4, *N* = 104) = 1.5204, *p* > 0.5] (Table S2).

## DISCUSSION

Previously tested techniques relying on maternal injections achieved in *N. vitripennis* a maximum efficiency of 0.8% genetically engineered offspring, or 13.3 genetically engineered wasps per 100 injections (Chaverra‐Rodriguez et al., 2020). Applying SYNCAS increased efficiency to 2.7% of offspring being genetically engineered, totalling 68 mutant wasps every 100 injections. Moreover, we obtained high heritability rates (83%–96%), higher than what was obtained via the use of BAPC alone (25%) or ReMOT (12%–75%) (Chaverra‐Rodriguez et al., 2020).

In our experiment, around one‐third of all injected females laid at least one genetically engineered embryo, suggesting that injecting a small number of wasps should be sufficient to obtain at least one mutant. For example, by injecting 10 virgin females, the probability of finding at least one genetically engineered male offspring should be above 98%.

SYNCAS is based on the synergistic action of saponins and BAPC (De Rouck et al., 2024). Although it is versatile and applicable to different arthropod species, saponin toxicity requires optimisation of the formulation composition. Indeed, Chaverra‐Rodriguez et al. (2020) reported detrimental effects on *N. vitripennis* survival, egg‐laying and offspring viability at saponin concentrations as low as 1 ng/μL, with only 20% of the females being alive 3 days after injection. In our experience, these negative effects have similar severity at higher concentrations (between 250 and 500 ng/μL), and other secondary effects reported by Chaverra‐Rodriguez and colleagues, such as induction of diapause in the offspring, are completely missing. Saponins are required to obtain efficient gene editing, as the addition of saponins at 150 ng/μL increases efficiency more than 34‐fold (0.08% vs 2.73%). Higher concentrations of saponins (300 ng/μL) did not increase the gene editing efficiency, yet they caused higher mortality and consequently less offspring. Therefore, we speculate that the maximum efficiency for this SYNCAS formulation has been reached, and lower concentrations of saponins might result in similar efficiency and cause lower lethality, reducing the number of injections necessary to generate a mutant.

Concentrations of the other SYNCAS components (RNP and BAPC) were not optimised here, although previous work in spider mites suggests that high RNP concentrations might be necessary to achieve high efficiency (De Rouck et al., 2024). Nonetheless, other researchers found that reducing the concentration of RNP from 4 to 2 μg/μL when performing DIPA did not result in an efficiency change (Zhang et al., 2024b). Moreover, the efficiency they reported is similar to the one we obtained with a 0 ng/μL saponin treatment, even though we used a concentration of Cas9 substantially higher (15 μg/μL). Therefore, we suggest that researchers planning to use this technique experiment with lowering the concentration of RNP when using the SYNCAS formulation.

The efficiency we reported for the mutation of c*innabar* should apply to other *N. vitripennis* target genes. Yet, for genes that do not have a known/visible phenotype, screening might remain hard even with efficiencies above 2.5%. To ease this task, one might consider co‐injecting Cas9‐RNP against a visible marker (e.g. *cinnabar*) and the desired target, as was already done for other species modified with SYNCAS (De Rouck et al., 2024). Zhang, Singh, et al. (2024) found that simultaneously injecting *N. vitripennis* mothers with two sgRNAs targeting sites at a few hundred base pair distances often caused a large deletion, suggesting that two distinct cutting events happened. Moreover, the genetically engineered offspring are not uniformly distributed among the hosts (Figure S2), and hosts containing one genetically engineered embryo are likely to contain additional ones. Thus, screening for mutants of the gene of interest only in broods having a mutant for a visible marker would alleviate the workload, both because the wasp mutant for the marker is likely a double mutant, and because another wasp in the same brood may carry the mutation of interest.

The fruitful application of SYNCAS to *N. vitripennis* opens the doors to its use for other Hymenoptera and other holometabolan insects. The insect species previously targeted with SYNCAS have ovariole structures different from those of *N. vitripennis*, and this might influence how nutrients and molecules from the haemolymph, including SYNCAS components, reach the developing embryos: *Nezara viridula* and *Bemisia tabaci* have teletrophic meroistic ovarioles (Fortes et al., 2011; Guo et al., 2016), *Frankiniella occidentalis* has neo‐panoistic ovarioles (Choi & Kim, 2022), whereas *N. vitripennis* has meroistic ovarioles, similarly to most Holometabola (Klowden & Palli, 2023). Therefore, our results show that SYNCAS could be a highly efficient technique for genetic engineering in other Holometabola with similar ovary structure.

In conclusion, we have demonstrated the efficiency of SYNCAS in the genetic engineering of another arthropod of which the embryos are difficult to manipulate, and we urge scientists working on other parasitoid species to exploit this powerful technique.

## EXPERIMENTAL PROCEDURES

We conducted experiments to examine the impact of two key factors on SYNCAS efficiency: the concentration of saponins and the time after oviposition when a female lays the highest number of genetically engineered eggs. An overview of the experimental procedure is provided in Figure S3.

### Insect strains and culturing

*Nasonia vitripennis* (strain AsymCx) wasps were held at 25°C and provided daily for 4 h with two hosts (*Calliphora vomitoria* pupae) to synchronise the age of the offspring. Ten days after oviposition, female *N. vitripennis* pupae were collected from the hosts and placed in a tube until eclosion. This was done to ensure that the females were virgins and capable of laying only haploid eggs. The wasps that were eclosed within an 8‐h window were transferred to another tube and were provided with 50% honey in water and fresh hosts to allow feeding. If not otherwise specified, 24 h later, the wasps were injected with treatment solutions.

### General aspects of injection

Injections were performed under a stereomicroscope, using a capillary pulled needle (borosilicate glass capillaries, 3.5″ Drummond #3–000‐203‐G/X), and a Femtojet 4i (Eppendorf, 5252000021). Females were anaesthetised on a CO_2_ pad and injected in the abdomen, ventral side, between sternites 3 and 4. Each wasp received around 200 nL of solution. The wasps were immediately provided with one host. Females were then moved to an incubator at 25°C and a 16/8 h light/dark cycle. The process was performed by two people, allowing for the injection of ~90 wasps per hour.

### Saponin toxicity

To test saponin toxicity, we injected solutions with 0, 31, 61, 125, 250, 500 and 1000 ng/μL of saponin (Merck, SAE0073) in PBS (Oxoid, BR0014G). Around 30 females per treatment were housed and injected as previously described and provided immediately with three hosts and placed in an incubator at 25°C and a 16/8 h light/dark cycle. The survival rate was checked at 24 and 48 h post‐injection, while the parasitisation rate was calculated after 20 days.

### SYNCAS mixture preparation

After the establishment of saponin toxicity, we tested whether saponins and BAPC would increase the gene editing efficiency of Cas9 in *N. vitripennis*.

Recombinant *Streptococcus pyogenes* Cas9 protein (Alt‐R® S.p. Cas9 Nuclease V3) was purchased from Integrated DNA Technologies (Leuven, Belgium) at a custom concentration of 50 μg/μL.

A sgRNA with protospacer sequence 5′‐GGAGCTTGTTCAGATGGGTT‐3′, specific to the eye‐pigmentation gene *cinnabar* was used, as it was previously demonstrated to work in vivo (Li et al., 2017). Single guide RNAs (sgRNAs) were ordered from IDT (ALT‐R CRISPR Cas9 sgRNA, 10 nmol). The sgRNAs were dissolved in nuclease‐free water to a final concentration of 10 μg/μL.

CRISPR/Cas9 Ribonucleoprotein particles (RNPs) were prepared by mixing 3 μL of 50 μg/μL Cas9 nuclease with 5 μL of 10 μg/μL sgRNA. The mixture was incubated for 10 min at room temperature. After incubation, 1 μL of BAPC at 10 μg/μL (BAPtofect®‐25, Phoreus Biotech, Olathe, US), and 1 μL of saponins (either 0, 1.5 or 3 μg/μL) were added, in this order. Based on the manufacturer's instructions and previous works (De Rouck et al., 2024), the dose of BAPC was determined by the amount of sgRNA used where BAPC was added in a sgRNA:BAPC mass ratio of 5:1. A 30‐min incubation on ice followed. Finally, the injection mix was centrifuged at 4 °C for 10 min at 20,000 *g* and kept on ice until use. The final concentrations of each component in the different mixtures used were 15 μg/μL Cas9, 5 μg/μL sgRNA, 1 μg/μL BAPC and a variable concentration of saponins (0, 150 or 300 ng/μL).

### Injections of SYNCAS mixtures

Between 45 and 90 virgin females were injected per treatment; each wasp was injected with ~200 nL of solution. Except when otherwise specified, the wasps were immediately placed in single tubes and provided with a host. After a period of 24 h, the hosts were collected in individual tubes labelled with a female‐specific code, and a second host was provided to the surviving females. After 24 h, the hosts were similarly collected, and surviving females were provided with three hosts. They were not re‐hosted after this point. This setup allowed us to define the optimal time point to screen for genetically engineered offspring.

### Offspring screening for gene editing events

All G0 red‐eyed males were collected in single tubes labelled with information regarding the mother's ID and time window of egg laying. Before being euthanised for DNA extraction and testing, red‐eyed males were put in individual tubes with three virgin females, which were then provided with 3–5 hosts. Ten days later, five G1 female pupae per mating were put in a single tube and upon emergence were provided with hosts. The resulting all‐male G2 was screened for the presence of red‐eye offspring as that would confirm the heritability of the mutation.

DNA was extracted by placing G0 red‐eye males in PCR tubes with 200 μL of freshly prepared 1.25% NH_4_OH (following Wielinga et al., 2006) (following Wielinga et al., 2006). Tubes were heated at 99°C for 20 min, briefly centrifuged (10–30 s), then reheated with open lids at 90°C until ~150 μL remained (~20 min). After another brief centrifugation, samples were stored at 4°C. The procedure was conducted in a fume hood. Each sample was used in *cinnabar* PCR amplification (M0492S, NEB Q5 polymerase) using forward (CTCTACGAGTACCGCTCAGG) and reverse (TGCAAGATCGAATCGTAACGC) primers, yielding ~520 bp amplicons. The amplicons were loaded on a 1% agarose gel to confirm specific and equal amplification and were then pooled per SYNCAS treatment and purified (28104, QIAGEN QIAquick PCR Purification Kit). Finally, amplicons were mass‐sequenced using Nanopore sequencing (Premium PCR sequencing, Plasmidsaurus, USA). Sequence alignments and KO allele frequencies were then analysed using the dedicated tools in Geneious Prime 2025.0.3.

## AUTHOR CONTRIBUTIONS

**Filippo Guerra:** Investigation; writing – original draft; formal analysis; data curation; visualization; writing – review and editing; conceptualization; methodology; validation. **Sander De Rouck:** Writing – review and editing; methodology; conceptualization; investigation; validation. **Eveline C. Verhulst:** Funding acquisition; resources; supervision; project administration; writing – review and editing; validation.

## FUNDING INFORMATION

This publication is part of the Open Competition ENW project with file number OCENW.M.22.140, which is financed by the Dutch Research Council (NWO). This work was also supported by ERC Proof of Concept [Grant 101123162] and Research Foundation Flanders (FWO) [Grant G035420N].

## CONFLICT OF INTEREST STATEMENT

The authors declare to have no conflict of interest.

## Supporting information


**Data S1.** Supporting information.

**Figure S1.** Supporting information.

**Figure S2.** Supporting information.

**Figure S3.** Supporting information.

**Table S1.** Supporting information.

**Table S2.** Supporting information.

## Data Availability

The data that support the findings of this study are openly available in Figshare, and are accessible at https://doi.org/10.6084/m9.figshare.28677017. (Guerra et al., 2025) Data can be used under licence CCBY 4.0. [Correction added on 09 August 2025 after first online publication: The Data Availability Statement has been updated.]
