# Supplementary material for: SYNCAS‐mediated CRISPR‐Cas9 genome editing in the Jewel wasp, Nasonia vitripennis
Source: Insect Mol Biol. 2025 Jul 17;35(1):48–55. doi: 10.1111/imb.70002 (PMC12779185; doi:10.1111/imb.70002)
Supplement: Supplementary file 1 — Data S1. Supporting information. [file IMB-35-48-s001.docx]

**Supplementary**

**Supplementary Table 1: Effects of saponin injections on *N. vitripennis* survival and oviposition ability.** A twofold dilution series was tested for effects on the survival and oviposition rate of injected wasps. The estimated dose that would result in the desired oviposition rate of 70% is around 150 ng/μl. h P.I. = hours post-injection.

| Saponin [ng/μl] | # Wasps injected | Alive 24h P.I. | Alive 48h P.I. | Ovipositing |
| --- | --- | --- | --- | --- |
| 0 | 34 | 33 | 33 | 33 (97%) |
| 31 | 33 | 33 | 32 | 32 (97%) |
| 63 | 20 | 20 | 19 | 19 (95%) |
| 125 | 29 | 28 | 21 | 23 (79%) |
| 250 | 34 | 14 | 13 | 13 (38%) |
| 500 | 27 | 5 | 3 | 2 (7.4%) |
| 1000 | 33 | 2 | 0 | 1 (3%) |

**Supplementary Table 2:** **SYNCAS formulations and injection time effects on mutation transmission rate.** Different treatments result in *cinnabar* mutants that can transmit the mutation to the next generations: in particular, they do not differ in mutation transmission rate, suggesting the level of chimerism is similar between treatments. The *chi*-square statistic is 1.5204. The *p*-value is > 0.67. The differences between groups do not deviate significantly from what could be expected by chance alone.

| **Chi-square calculation for mutation transmission rate** | | | |
| --- | --- | --- | --- |
|  | Have GM offspring | No GM offspring | ***Row Totals*** |
| Day 1, 150 ng/μl sap | 28  (27.40)  [0.01] | 2  (2.60)  [0.14] | 30 |
| Day 1, 300 ng/μl sap | 5  (5.48)  [0.04] | 1  (0.52)  [0.45] | 6 |
| Day 4, 150 ng/μl sap | 40  (41.11)  [0.03] | 5  (3.89)  [0.31] | 45 |
| Day 1, delayed ,150 ng/μl sap | 22  (21.01)  [0.05] | 1  (1.99)  [0.49] | 23 |
|  |  |  |  |
| ***Column Totals*** | 95 | 9 | **104**  **(Grand Total)** |


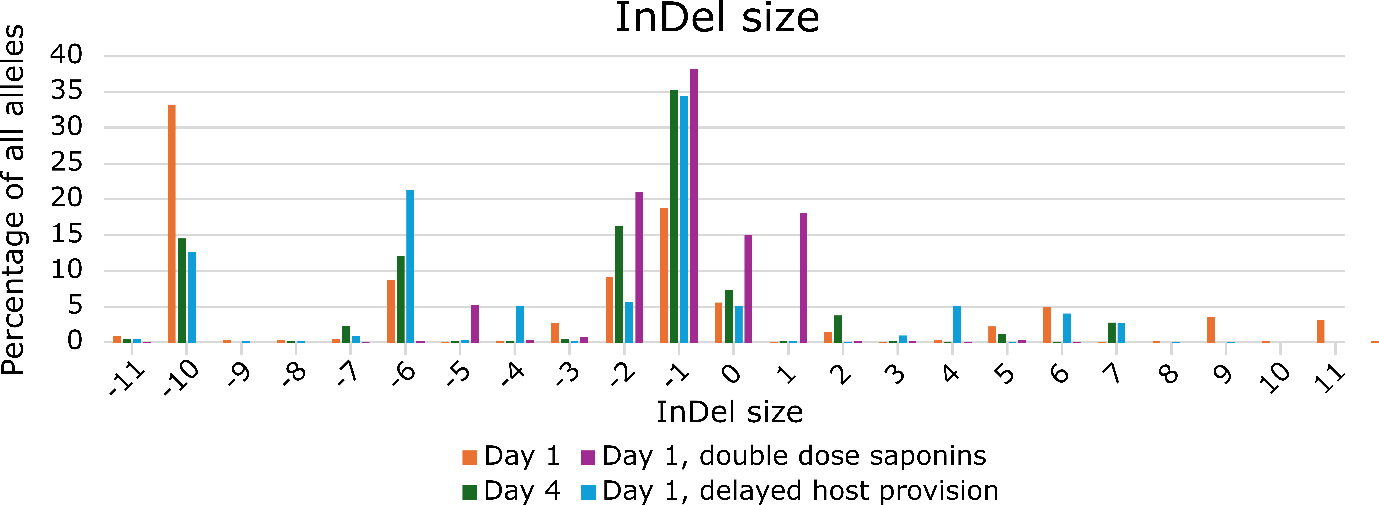


**Supplementary Fig. 1: Size of insertions and deletions (InDel) around the Cas9 cutting site.** The InDel size is dependent on the DNA repair mechanism and multiple mutations are generated with InDel size between -10 and +11 base pairs. In orange, is the analysis of mutant offspring of one-day-old wasps injected with SYNCAS at 150 ng/μl saponins (4781 reads from 38 mutants); in green is the analysis of four-day-old wasps injected with SYNCAS at 150 ng/μl saponins (4549 reads from 52 mutants); in purple is the analysis of one day old injected with SYNCAS at 300 ng/μl saponins (3784 reads from 6 mutants); in blue is the analysis of one day old injected with SYNCAS at 150 ng/μl saponins and delaying host provision by 24 hours (4684 reads from 37 mutants). Graph was generated with Microsoft Excel (<https://office.microsoft.com/excel>). The raw sequencing data and the classification of gene editing events is available at https://doi.org/10.6084/m9.figshare.28677017


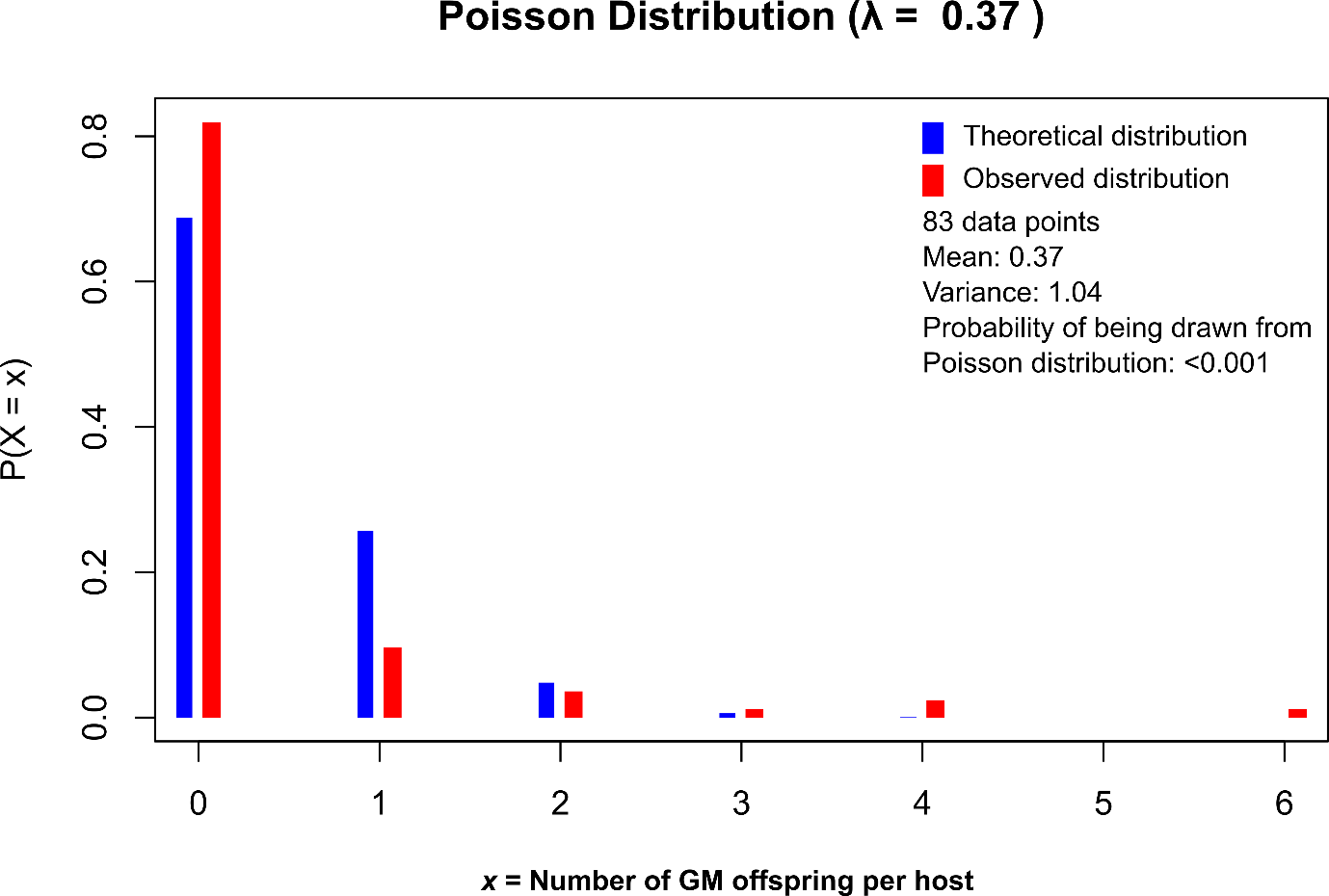


**Supplementary Fig. 2: Distribution of genetically modified offspring number in parasitized hosts.** When screening for a mutation, the probability of finding a host harbouring at least one GM embryo is 18%. The probability of finding a second mutated wasp in said hosts is 47%. In particular, the offspring are not distributed as expected by chance, since they would follow a Poisson distribution. This suggests that co-injecting Cas9 RNPs targeting a visible marker (such as *cinnabar*) and a gene of interest (GOI) lacking an associated phenotype would aid the screening and identification of mutants as broods containing phenotypic mutants are more likely to contain mutants in the GOI. The data shown in the graph are relative to the egg clutches from injections in one-day-old females with 150 ng/ μl saponins and collected between 0 and 48 hours post-injection. Image generated with R v4.4.3 (R Core Team, 2021).


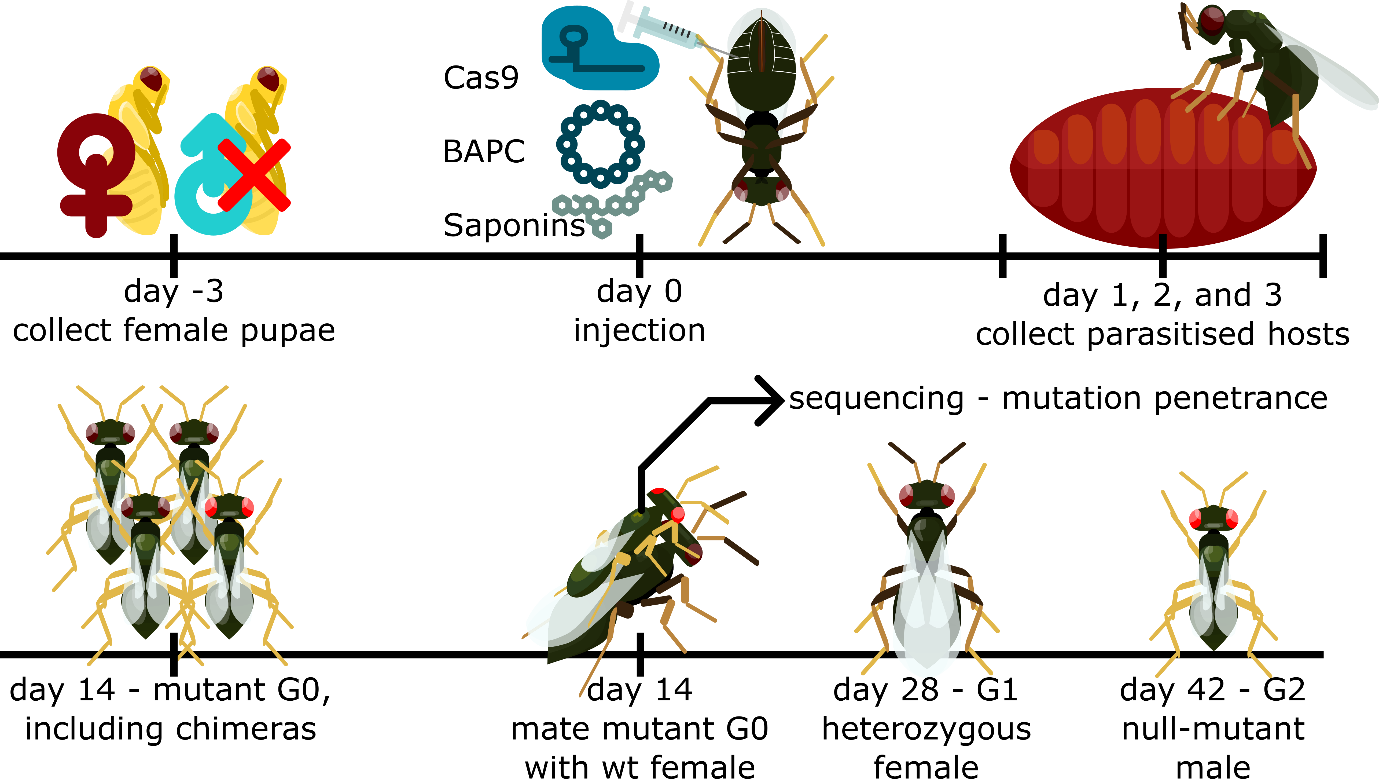


**Supplementary Figure 3: An overview of the methodology to obtain *Nasonia* *vitripennis* mutants using SYNCAS technology.** Three days before injection, female *N. vitripennis* pupae of the same age are collected to obtain synchronized emergence of adults and ensure they are not mated and therefore capable of laying only unfertilized haploid eggs that develop into males. Wasps are injected between the third and the fourth abdominal segment with a mixture of Cas9 ribonucleoparticle, BAPC, and saponins. They are immediately provided with a host (blowfly pupae), which is collected and replaced every 24 hours. Incubation at 25 °C allows the G0 male offspring to emerge in two weeks. Screening for mutants is done by visual inspection; the progeny might contain phenotypically mutant chimaeras. To test for the heritability of the mutation and obtain null-mutant lines, the mutant males are mated with wild-type females for 24 hours. This possibly yields heterozygous G1 females, which lay null-mutant and wild-type eggs. By mating the heterozygous females with their son it is possible to obtain homozygous females and stable mutant lines. Genotypic testing via PCR amplification and sequencing of G0 informs on the penetrance of the mutation. Image generated with Inkscape v1.4 (https://inkscape.org/).
