# Supplementary figures and images for: SYNCAS‐mediated CRISPR‐Cas9 genome editing in the Jewel wasp, Nasonia vitripennis
Source: Insect Mol Biol. 2025 Jul 17;35(1):48–55. doi: 10.1111/imb.70002 (PMC12779185; doi:10.1111/imb.70002)

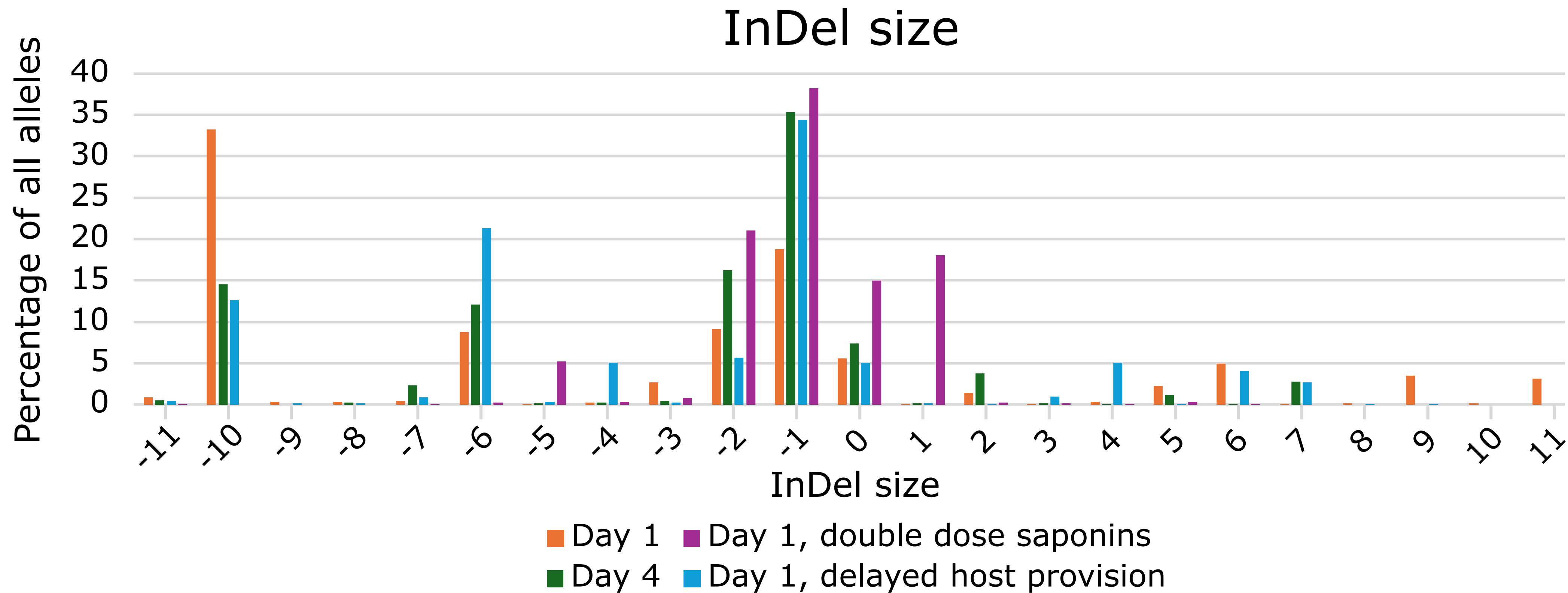

Supplement: Supplementary file 2 — Figure S1. Supporting information. [file IMB-35-48-s003.pdf]

## Poisson Distribution ( $\lambda = 0.37$ )

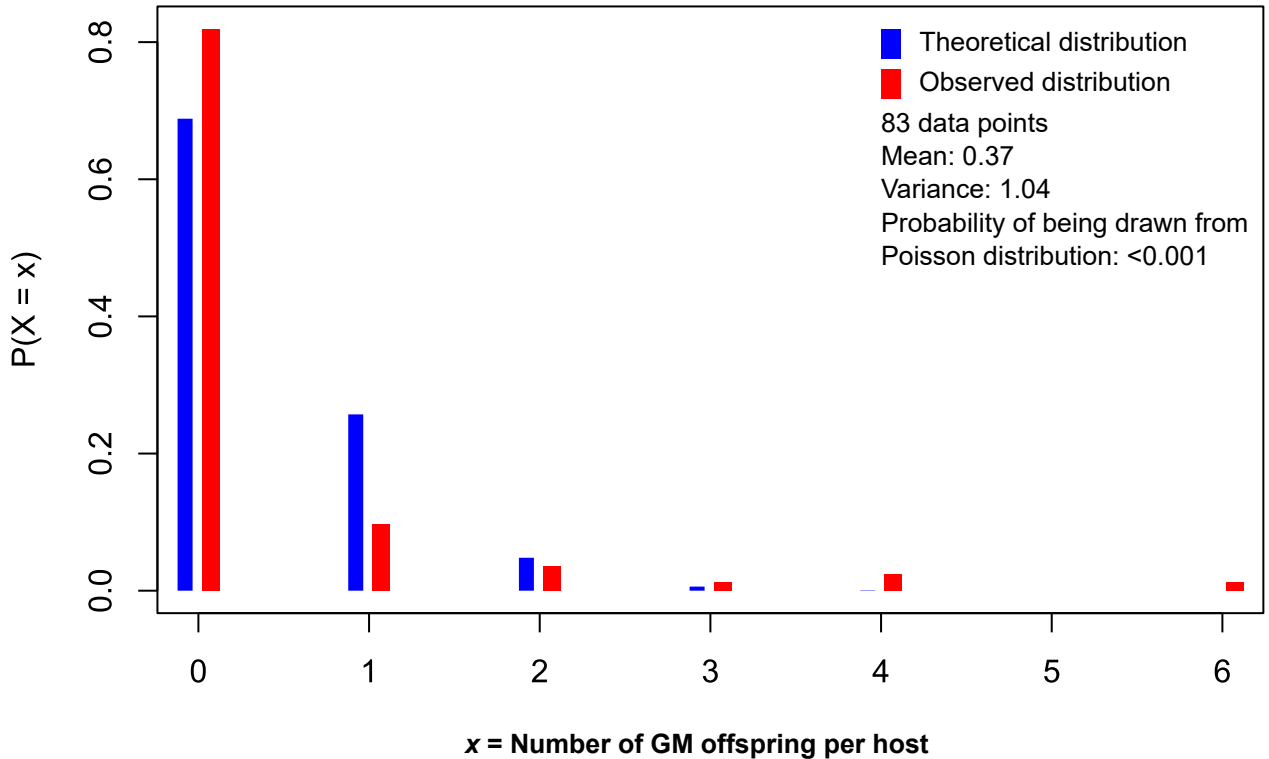

Supplement: Supplementary file 3 — Figure S2. Supporting information. [file IMB-35-48-s006.pdf]

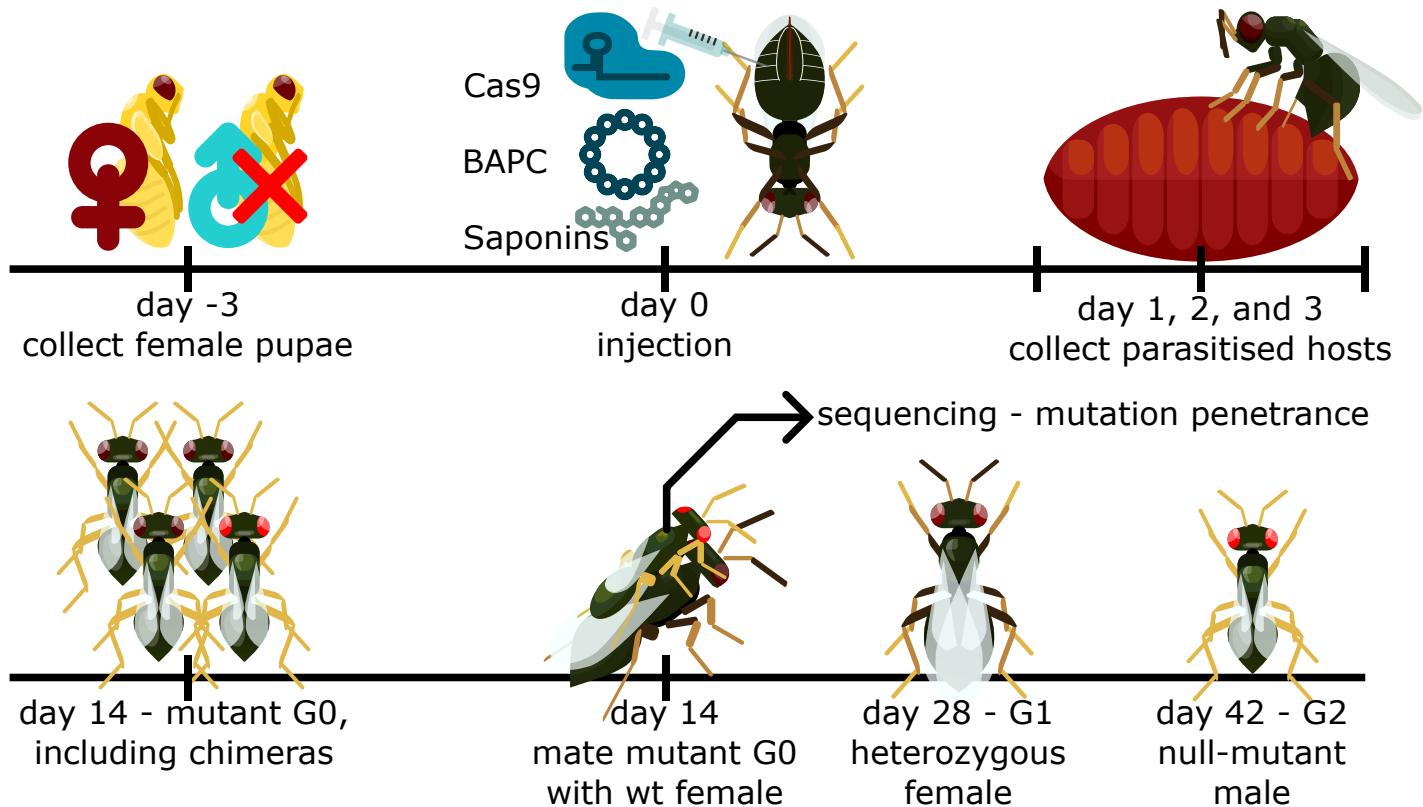

Supplement: Supplementary file 4 — Figure S3. Supporting information. [file IMB-35-48-s004.pdf]
