# Supplementary material for: SYNCAS‐mediated CRISPR‐Cas9 genome editing in the Jewel wasp, Nasonia vitripennis
Source: Insect Mol Biol. 2025 Jul 17;35(1):48–55. doi: 10.1111/imb.70002 (PMC12779185; doi:10.1111/imb.70002)
Supplement: Supplementary file 5 — Table S1. Supporting information. [file IMB-35-48-s005.docx]

| Saponin [ng/μl] | # Wasps injected | Alive 24h P.I. | Alive 48h P.I. | Ovipositing |
| --- | --- | --- | --- | --- |
| 0 | 34 | 33 | 33 | 33 (97%) |
| 31 | 33 | 33 | 32 | 32 (97%) |
| 63 | 20 | 20 | 19 | 19 (95%) |
| 125 | 29 | 28 | 21 | 23 (79%) |
| 250 | 34 | 14 | 13 | 13 (38%) |
| 500 | 27 | 5 | 3 | 2 (7.4%) |
| 1000 | 33 | 2 | 0 | 1 (3%) |
