# Supplementary material for: SYNCAS‐mediated CRISPR‐Cas9 genome editing in the Jewel wasp, Nasonia vitripennis
Source: Insect Mol Biol. 2025 Jul 17;35(1):48–55. doi: 10.1111/imb.70002 (PMC12779185; doi:10.1111/imb.70002)
Supplement: Supplementary file 6 — Table S2. Supporting information. [file IMB-35-48-s002.docx]

| **Chi-square calculation for mutation transmission rate** | | | |
| --- | --- | --- | --- |
|  | Have GM offspring | No GM offspring | ***Row Totals*** |
| Day 1, 150 ng/μl sap | 28  (27.40)  [0.01] | 2  (2.60)  [0.14] | 30 |
| Day 1, 300 ng/μl sap | 5  (5.48)  [0.04] | 1  (0.52)  [0.45] | 6 |
| Day 4, 150 ng/μl sap | 40  (41.11)  [0.03] | 5  (3.89)  [0.31] | 45 |
| Day 1, delayed ,150 ng/μl sap | 22  (21.01)  [0.05] | 1  (1.99)  [0.49] | 23 |
|  |  |  |  |
| ***Column Totals*** | 95 | 9 | **104**  **(Grand Total)** |
